# Enhanced lithium storage performance of V_2_O_5_ with oxygen vacancy

**DOI:** 10.1039/c8ra07326k

**Published:** 2018-11-26

**Authors:** Yinlu Sun, Zhiping Xie, Yanwei Li

**Affiliations:** College of Chemistry, Liaoning University Shenyang 110036 Liaoning China; Guangxi Key Laboratory of Electrochemical and Magneto-chemical Functional Materials, College of Chemistry and Bioengineering, Guilin University of Technology Guilin 541004 Guangxi China lywhit@126.com

## Abstract

Orthorhombic phase V_2_O_5_ nanosheets with a high V^4+^ content (V-V_2_O_5_) have been fabricated *via* a facile sol–gel method and freeze-drying technology followed with a vacuum annealing process. XPS tests demonstrated that the content of V^4+^ in the as-prepared V-V_2_O_5_ sample was 7.4%, much higher than that (4.7%) in the V_2_O_5_ sample annealed in air. Compared with the V_2_O_5_ annealed in air, the V-V_2_O_5_ sample exhibited better cycling stability, higher lithium storage activity, and smaller electrochemical reaction resistance when evaluated as a cathode active material for lithium ion batteries. For example, the specific capacity of the V-V_2_O_5_ and V_2_O_5_ electrodes after 100 cycles at 200 mA g^−1^ are 224.7 and 199.2 mA h g^−1^, respectively; after 200 cycles at 3 A g^−1^ are 150 and 136.7 mA h g^−1^, respectively.

## Abstract 2

Orthorhombic phase V_2_O_5_ nanosheets with a high V^4+^ content (V-V_2_O_5_) have been fabricated *via* a facile sol–gel method and freeze-drying technology followed with a vacuum annealing process.

## Graphical abstract

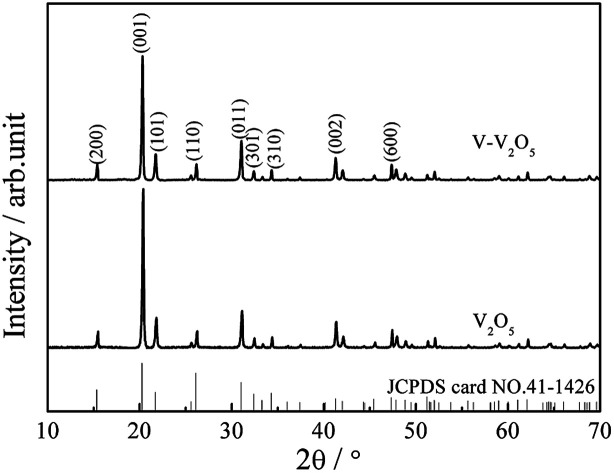

## 1. Introduction

Orthorhombic vanadium pentoxide (V_2_O_5_) has been widely studied in lithium ion battery cathode materials because of its typical lamellar crystal structure.^1^ Compared with conventional lithium ion battery cathode materials, V_2_O_5_ has much higher theoretical capacity (294 mA h g^−1^, when storing two Li^+^) than other materials. V_2_O_5_ also has the advantages of abundant resources, low cost, relatively simple preparation process and good safety.^2–4^ However, the drawbacks such as structure instability, low electronic and ionic conductivity and slow electrochemical kinetics drastically reduce its cycling stability and rate capability, which has become the main restrictions to its practical application in the cathode materials of lithium ion batteries.^5,6^ To overcome these drawbacks, numerous efforts have been carried out. Some researches mainly concentrate on modifier materials such as various nanostructures V_2_O_5_,^7,8^ aliovalent-ion doped V_2_O_5_,^6,9^ V_2_O_5_ coated with carbon materials or other compounds.^10,11^ These modifiers showed enhanced electrochemical kinetics, specific capacity and rate performance than the pristine V_2_O_5_ due to their small size and large surface area, which could greatly increase the contact area between actives materials and electrolyte, shorten the diffusion pathways of Li^+^, and relax the mechanical stress associated Li^+^ intercalation/deintercalation. However, it should be noted that these modifications are complex, difficult to control and costly.

The heat treatment atmosphere has great influence on the valence state of V element in V_2_O_5_. Generally, when being heat treated in an oxidation atmosphere (such as air and O_2_ atmosphere), the V^4+^/V^5+^ ratio and O vacancies in the final V_2_O_5_ product are very small; when being heat treated in inert gas (such as Ar atmosphere), and reduction atmospheres (such as H_2_ atmosphere), the V^4+^/V^5+^ ratio and O vacancies in the final V_2_O_5_ product are high, and the V_2_O_5_ phase can even transform to VO_2_ phase in strong reduction atmosphere. These O vacancies in V_2_O_5_ can provide more active sites for the embedding of Li^+^, and increase the specific discharge capacity of the materials. O vacancies can also increase the electronic and ionic conductivity of the materials and promote the lithium storage kinetics.^12–14^ However, the preparation process of V_2_O_5_ with O vacancy is complex and consumptive, and the electrochemical lithium storage capacity is not satisfactory. It is found that under the condition of oxygen depletion, oxygen can easily escape from the lattice due to the breakage of the V=O bond, resulting in the formation of multiple O vacancies in V_2_O_5_ materials.

Herein, we prepared orthorhombic phase V_2_O_5_ nanosheets with a high V^4+^ content and O vacancies (denoted as V-V_2_O_5_) by a facile sol–gel method combined with freeze drying technique followed with annealing in vacuum. The microstructure of V-V_2_O_5_ was analyzed by physical characterizations, and the electrochemical properties of V-V_2_O_5_ were evaluated as potential cathode materials for lithium ion batteries (LIBs). The results show that the electrode material has good lithium storage activity, cyclic stability and high current discharge performance.

## 2. Materials and methods

### 2.1 Synthesis of V-V_2_O_5_

All chemical reagents were of analytical purity and used without further purification. The synthesis of V-V_2_O_5_ is as following: firstly, commercialized V_2_O_5_ powder (C-V_2_O_5_) was added into de-ionized (DI) water and 30 wt% H_2_O_2_ to obtain a solution with a V_2_O_5_ concentration of 0.3 M and *n*(H_2_O_2_) : *n*(V) = 8 : 1. The resulting solution was stirred for 15 min at room temperature. Sonicated for 10 min, the obtained solution was diluted with DI to *C*_V_ = 0.056 M. And then the solution was sonicated for about 1 h until it turned into brownish red V_2_O_5_ gel. The gel was stored overnight and diluted with DI water and fully stirred to form a brick-red coloured, transparent sol with *C*_V_ of 0.028 M. Secondly, this solution was pre-frozen in a refrigerator for 1 day and then the solvent (water) in the frozen sample was removed using a freeze dryer (FD-1A-50, Boyikang Corp., Beijing, China) under a vacuum at 50 °C for 2 days to get the V_2_O_5_ precursor. Thirdly, the V_2_O_5_ precursor was annealed in vacuum at 400 °C for 1 h and then annealed at 400 °C in the air for 1 h to form V_2_O_5_ containing O vacancy (denoted as V-V_2_O_5_). For comparison, the V_2_O_5_ precursor was also annealed in air for 2 h and the final product is denoted as V_2_O_5_.

### 2.2 Material characterizations

The crystal structure of the prepared samples was determined on X'Pert^3^ diffractometer (PANalytical, Netherlands) with a Cu K_α_ as radiation source (*λ* = 1.54056 °A) in a 2*θ* range of 10–70°. The morphology of the prepared samples was characterized on a field-emission scanning electron microscopy (FE-SEM) (Hitachi, SU-5000) and transmission electron microscope (TEM, JEOL JEM-2100F). The chemical composition and the valence state of V in the prepared samples were analyzed by X-ray photoelectron spectroscopy (XPS) spectrometer (ESCALAB 250Xi) using monochromic Al K_α_ excitation.

### 2.3 Electrochemical measurements

The electrochemical performances of the samples were tested using CR 2016 coin-type cell with metallic lithium as the anode and polypropylene (PP) film as the separator. The cathodes were fabricated by mixing V-V_2_O_5_, acetylene black, and poly(vinyldifluoride) (PVDF) with a weight ratio of 7 : 2 : 1 in *n*-methyl-2-pyrrolidone (NMP) solvent. The resulting slurry was then uniformly spread on an aluminium foil current collector. The cathodes were dried at 80 °C for 12 h in an oven and then punched into small disks with a diameter of 15 mm. The thickness of the electrode was about 15 μm and the mass loading of the active material was about 1.0 mg cm^−2^. The electrolyte was 1 M LiPF6 in EC/DMC/DEC (1 : 1 : 1 by weight). The cells were galvanostatically charged and discharged between 2.0 and 4.0 V (*vs.* Li/Li^+^) using LANDCT2001A battery tester at room temperature. Cyclic voltammetry (CV) and electrochemical impedance spectroscopy (EIS) measurements were carried out on an electrochemical workstation (CHI 760). The CV test was performed between 2.0 and 4.0 V at a scan rate of 0.2 mV s^−1^. The EIS was measured was performed in the frequency range from 0.01 Hz to 100 kHz at the open circuit voltage (OCV) after given discharge/charge cycles with 5 mV voltage amplitude.

## 3 Results and discussions

### 3.1 Structure characterization

**Fig. 1:**
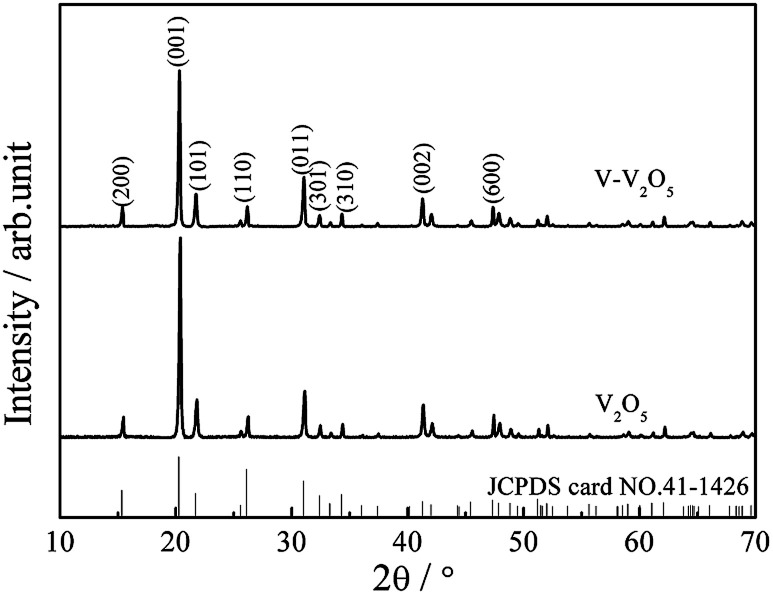
XRD patterns of the V-V_2_O_5_ and V_2_O_5_ samples.

Fig. 1 presents the XRD patterns of the pure V_2_O_5_ and V-V_2_O_5_ samples. Both pure V_2_O_5_ and V-V_2_O_5_ samples show a single orthorhombic V_2_O_5_ phase (JCPDS card no. 41-1426) without detectable secondary phase. The peaks around 2theta = 15.49°, 20.35°, 26.23° and 31.09° correspond to (200), (001), (101), (110) diffraction peak of the orthogonal phase V_2_O_5_. By careful observation, it can be found that there is obvious difference of the relative intensities of (001), (110) and (011) diffraction peaks for the two samples. For V-V_2_O_5_, the intensity ratio between (001) and (110) diffractions and the ratio between (001) and (011) diffractions are 6.2 and 3.3, respectively, which are smaller than those (9.5 and 4.3) of pure V_2_O_5_. This indicates that the V-V_2_O_5_ exposed more facets than the pure V_2_O_5_. The lattice constants of samples were calculated based on the XRD patterns in Fig. 1 by jade software. For V_2_O_5_, calculated lattices constants are *a* = 11.484 Å, *b* = 3.556 Å, *c* = 4.357 Å; for V-V_2_O_5_, the calculated lattice constants are *a* = 11.513 Å, *b* = 3.564 Å, *c* = 4.372 Å. It can be seen that the V-V_2_O_5_ shows slight lattice expansions as compared to that of V_2_O_5_. It is known that radius of V^4+^ is larger than that of V^5+^. Therefore, the lattice expansion can be attributed to the increased V^4+^ content in V-V_2_O_5_ sample (Fig. 3).

**Fig. 2:**
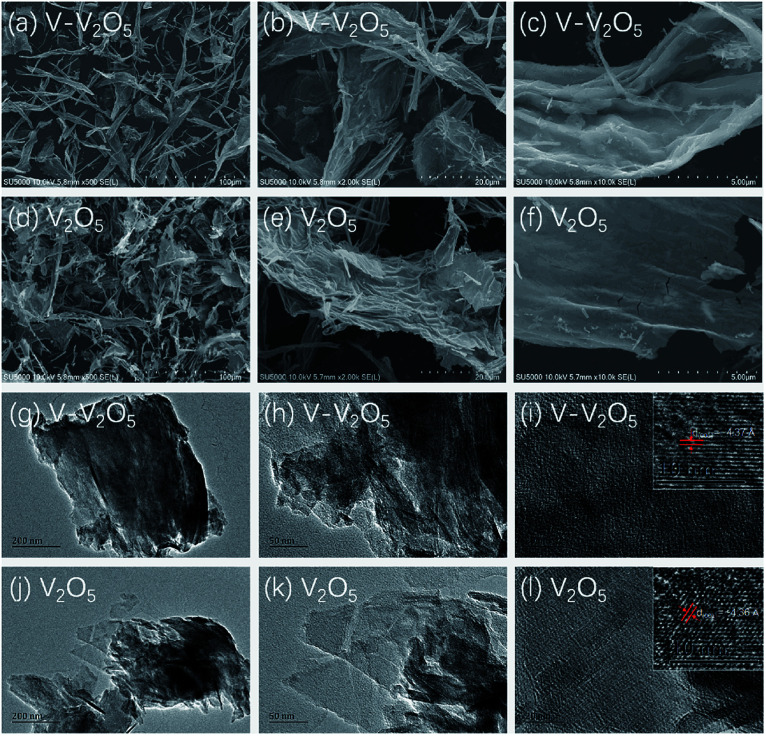
FESEM images of the V-V_2_O_5_ (a–c) and V_2_O_5_ (d–f) samples; TEM images of the V-V_2_O_5_ (g–i) and V_2_O_5_ (j–l) samples.

Fig. 2 gives the SEM and TEM images of the pure V_2_O_5_ and V-V_2_O_5_ samples. It can be seen that both V-V_2_O_5_ and V_2_O_5_ are 2D sheet-like morphologies. By observing the high magnification SEM and TEM images, the surface of the V-V_2_O_5_ has a lot of deeper ravines and the surface fluctuates greatly (Fig. 2c, g and h), while V_2_O_5_ is a flat sheet with cracks attributed to the mechanical force in the sample preparation process (Fig. 2f, j and k). Compared with pure V_2_O_5_, the coarse surface of V-V_2_O_5_ is more effective to inhibit the stacking of sheets, store more electrolyte, offer larger material–electrolyte contact area, and relax the mechanical strain generated upon the lithium ion intercalation/deintercalation cycles. The lattice fringe (inset of Fig. 2i and l) of about 4.36 Å can be assigned to the (001) plane of orthorhombic V_2_O_5_, consistent with the XRD patterns shown in Fig. 1.

**Fig. 3:**
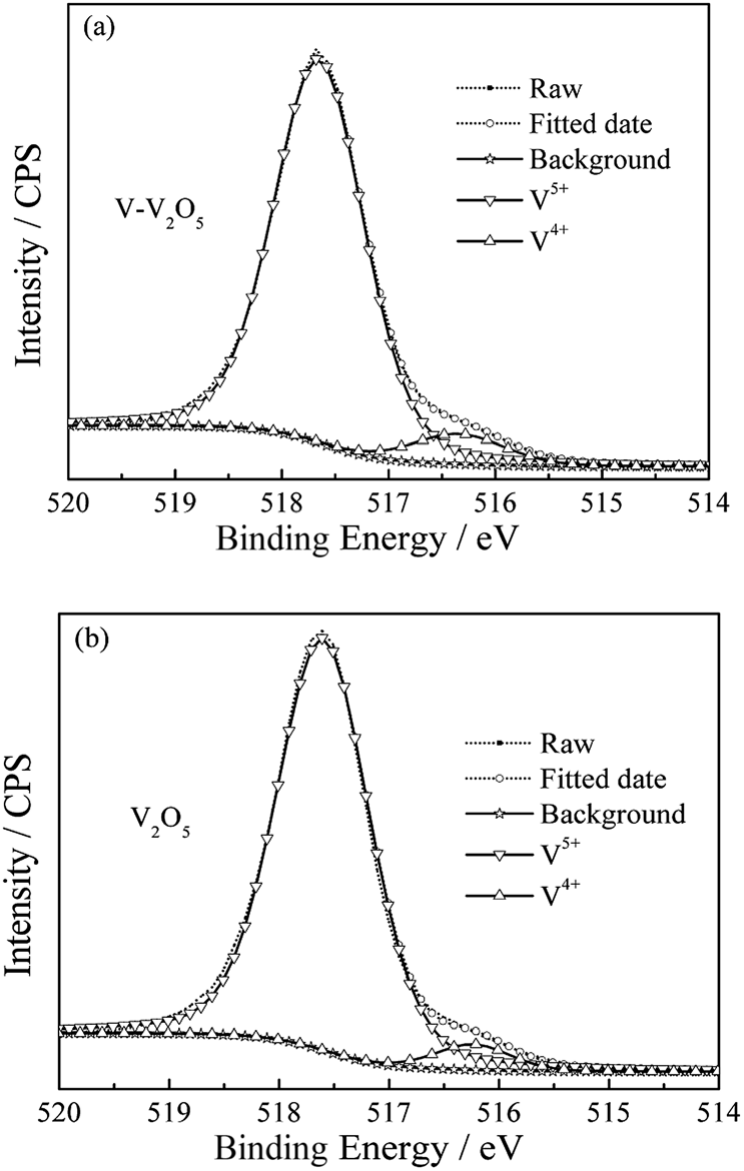
XPS spectra on V2p_3/2_ of (a) V-V_2_O_5_ and (b) V_2_O_5_ samples.

XPS was carried out to investigate the oxidation state of vanadium and O vacancies in V-V_2_O_5_ and V_2_O_5_ samples. The V2p_3/2_ spectra shown in Fig. 3 compose of two components locating at the binding energy values of 517.6 and 516.3 eV, which are associated with two formal oxidation degrees, V^5+^ and V^4+^.^15,16^ From the area ratio of the fitted spectrum of V^5+^ and V^4+^, the molar ratios of V^4+^/(V^4+^ + V^5+^) in V-V_2_O_5_ and V_2_O_5_ are 7.4% and 4.7%, respectively. According to charge neutrality of system, the formation of one O vacancy should corresponds the generation of two V^4+^. Thus, the O vacancies concentrations in V-V_2_O_5_ and V_2_O_5_ are calculated to be 3.7% and 2.35%, respectively. The increased of V^4+^ content in V-V_2_O_5_ can be attribute to the loss of lattice O in V_2_O_5_ during the annealing process in oxygen depletion condition (vacuum). The present of oxygen vacancies could leave more open voids, which will provide more migration paths for the fast Li^+^ diffusion and facilitate the reversible phase transitions of V_2_O_5_ during the Li^+^ insertion/extraction process.^12,17^ Furthermore, it has been suggested that the mixed valence V^4+^/V^5+^ could improve the electrical conductivity of the material due to the synergic activity in V_2_O_5_ materials.

### 3.2 Electrochemical performances

**Fig. 4:**
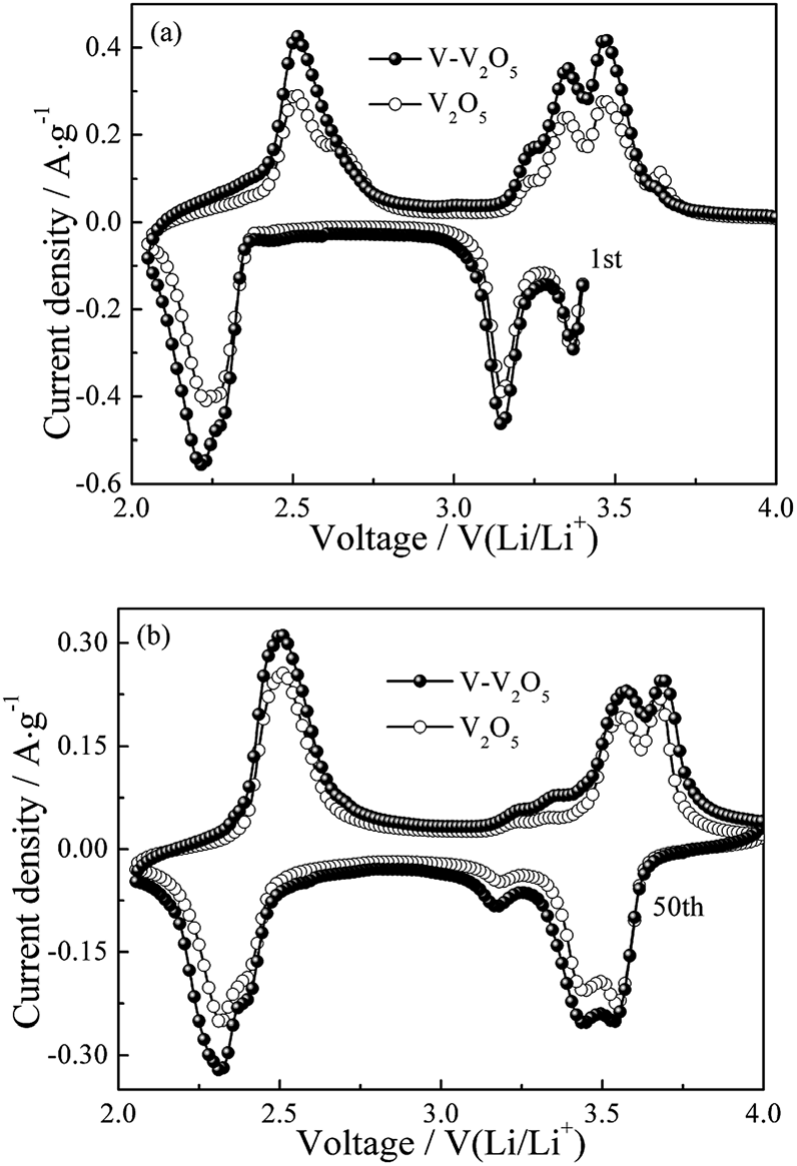
The (a) first and (b) fifth cycles of CV curves of V-V_2_O_5_ and V_2_O_5_ electrodes at a scan rate of 0.2 mV s^−1^.

Fig. 4 show the CV profiles of the first and the fifth cycles at a scan rate of 0.2 mV s^−1^ for the pure V_2_O_5_ and V-V_2_O_5_ electrodes, respectively. As seen from Fig. 4a, for pure V_2_O_5_ and V-V_2_O_5_ in the first cycle, three reduction peaks at 3.35, 3.10 and 2.20 V are observed, which correspond to the α/ε, ε/δ and δ/γ phase transitions, respectively. During the Li^+^ deintercalation process, four oxidation peaks are observed for two samples. The peak at 2.51 V corresponds to the γ/δ phase transition, the peak at 3.24 and 3.35 V correspond to the δ/ε phase transition, and the peak at 3.47 V corresponds to the ε/α phase transition.^18–20^ However, after the first intercalation process, the irreversible γ′ phase of V_2_O_5_ is indicated by the emergence of oxidation peak at 3.65 V and reduction peaks broadening at 2.22 V.^19,21^ After 50 cycles in Fig. 4b, the reduction peak currents of the V-V_2_O_5_ and V_2_O_5_ decrease at 3.16 V while increase at 3.54 V in the γ′ intercalation process; simultaneously, the two oxidation peaks corresponding to δ/ε phase transition decrease at 3.24 and 3.35 V while increase at 3.65 V. Additionally, the peak area of V-V_2_O_5_ is always greater than that of pure V_2_O_5_, which indicates that the V-V_2_O_5_ sample obtained by vacuum annealing has a higher lithium storage activity.

**Fig. 5:**
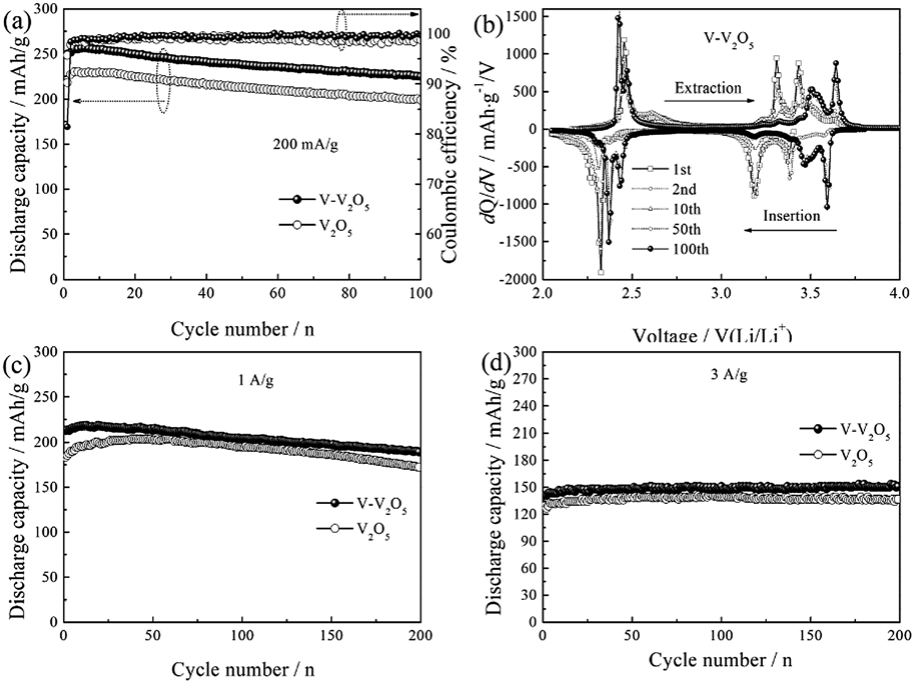
(a) Cycling performance and coulombic efficiencies of the V-V_2_O_5_ and V_2_O_5_ electrodes at a current density of 200 mA g^−1^; (b) differential specific capacity plots of the V-V_2_O_5_ at different cycle number at a current density of 200 mA g^−1^, cycling performance of V-V_2_O_5_ and V_2_O_5_ at a current density of (c) 1 A g^−1^ and (d) 3 A g^−1^, respectively.

Fig. 5a gives the cycling response of the V-V_2_O_5_ and pure V_2_O_5_ electrodes at a current density of 200 mA g^−1^. Obviously, V-V_2_O_5_ electrode exhibits higher discharge capacity than the pure V_2_O_5_ electrode. The maximum discharge specific capacities of V-V_2_O_5_ and V_2_O_5_ samples are 230.2 and 256.6 mA h g^−1^, respectively. The discharge specific capacities after 50 cycles are 213.1 and 237.9 mA h g^−1^, and after 100 cycles are 199.2 and 224.7 mA h g^−1^, respectively. The intercalation/deintercalation lithium capacity of V-V_2_O_5_ is always higher than that of V_2_O_5_. This change of discharge capacity is in accordance with the CV results (Fig. 4). The capacity retention of V-V_2_O_5_ electrode at 100^th^ cycle is 87.6%, superior to that reported in the literatures on the hydrogenated V_2_O_5_ prepared by the H_2_ reduction (84% at 100 mA g^−1^ and 100^th^ cycle),^13^ on the V_2_O_5_ nanorods prepared by the electrostatic spinning method (43% at 50 mA g^−1^ and 50^th^ cycle)^22^ and on the carbon coated V_2_O_5_ nanoparticles (82% at 29.4 mA g^−1^ and 30^th^ cycle),^10^ indicating an excellent capacity retention capability.

Fig. 5b presents the differential capacity corresponding to the cyclic performance of V-V_2_O_5_ in Fig. 5a. During the first discharge, the three peaks at 3.37, 3.2 and 2.32 V correspond to the α/ε, ε/δ and δ/γ phase transitions, respectively, in accordance with the reduction peaks in CV curve (Fig. 4a); during the charging process, the peak at 2.45 V corresponds to the γ/δ phase transition, the peak at 3.23 and 3.30 V correspond to the δ/ε phase transition, and the peak at 3.43 V corresponds to the ε/α phase transition, in accordance with the oxidation peaks in CV curve (Fig. 4a). With the increase of charge/discharge the phase transition between ε phase and δ phase decreases gradually, while the intensities of peaks at 3.57 V upon the lithium ion intercalation cycle and at 3.65 V upon the lithium ion deintercalation cycle are gradually increased. This observation is in good agreement with the CV results.

In order to further study the cyclic stability of V-V_2_O_5_ under high current, the cyclic properties of V-V_2_O_5_ and V_2_O_5_ samples were compared at 1 and 3 A g^−1^ current densities, respectively (Fig. 5c and d). At 1 A g^−1^, the discharge specific capacity of V-V_2_O_5_ and V_2_O_5_ rises first and then decreases, and the maximum capacities are 218.4 and 200.6 mA h g^−1^, respectively. The significantly improved capacity in the first few cycles during cycling measurement can be attributed the activation of electrode (the penetration of electrolyte and/or increase of active surface). The discharge specific capacities of V-V_2_O_5_ and V_2_O_5_ after 200 cycles are 189.3 and 172.4 mA h g^−1^, respectively. When the current density increases to 3 A g^−1^, the capacity of two electrodes increase gradually and remain stable. The maximum discharge specific capacities of V-V_2_O_5_ and V_2_O_5_ are 150 and 141.7 mA h g^−1^, respectively. The excellent lithium storage performance of V-V_2_O_5_ could be ascribed to the vacuum annealing process. Under the condition of oxygen poor, V_2_O_5_ easily loses the O in the crystal structure and forms the O vacancies. The O vacancies can help reduce V^5+^ to V^4+^, which increases the electronic and ionic conductivity of the material and also provide additional embedding position for the lithium ion.

**Fig. 6:**
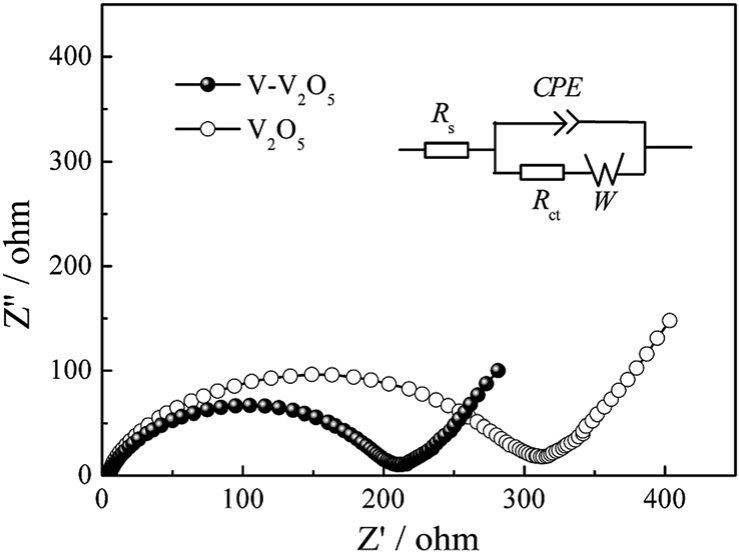
Nyquist plots of V-V_2_O_5_ and V_2_O_5_ electrodes.

The Nyquist impedance spectra of V-V_2_O_5_ and V_2_O_5_ are shown in Fig. 6, measured at fully discharged state under 200 mA g^−1^ in 100th cycles. The Nyquist plots were fitted using an analogical equivalent circuit. In this equivalent circuit, *R*_s_ corresponds to the equivalent series resistance (ESR) which contains all ohmic resistance due to the electrolyte and other parts of the system. Charge transfer resistance (*R*_ct_) represents the charge-transfer impedance at the electrode/electrolyte interface, corresponding to the semicircles in high-medium frequency scope. *W* refers to the Warburg impedance, corresponding to the inclined line in the low-frequency scope. A constant phase element (CPE) is used in the equivalent circuit instead of a pure capacitance due to the inhomogeneous surface of the working electrode. The solid line represents the impedance calculated using the equivalent circuit and the error between the experimental and fitting data was less than 1%. Note that the depressed semicircles related to the charge transfer in the high-medium frequency region and the angled straight line corresponding to the low frequency range can be effectively simulated. The fitting parameters of the semicircle can be calculated from the corresponding equivalent circuit. The *R*_ct_ values of V-V_2_O_5_ and V_2_O_5_ are 213.1 and 320.6 Ω, respectively. It evidently implies that V-V_2_O_5_ possesses higher electrochemical reaction kinetics, which is ascribed to its O vacancies facilitating lithium insertion/extraction.

## 4 Conclusions

The orthorhombic phase V_2_O_5_ (V-V_2_O_5_) nanosheet with O vacancies was prepared by a facile sol–gel method combined with freeze drying technique followed with annealing in vacuum. XRD analysis revealed that the relative intensities of (110) and (011) diffraction peaks for V-V_2_O_5_ were enhanced, and V-V_2_O_5_ showed slight lattice expansions due to O vacancies. FESEM demonstrated that V-V_2_O_5_ was 2D sheet-like morphology with coarse surface. XPS tests indicated that O vacancies induced more V^4+^ in V_2_O_5_. When used as cathode material for Li-ion batteries, the V-V_2_O_5_ exhibited much enhanced rate capability and cycling stability as compared to the pure V_2_O_5_ counterpart. The superior lithium storage performance of the V-V_2_O_5_ could be ascribed to the following reasons: coarse surface improves the specific surface area of the nanosheet, and increases the effective contact area between electrode and electrolyte; the predominantly exposed (110) and (011) crystal planes of V-V_2_O_5_ provide channels for facile Li^+^ intercalation and deintercalation, which contributes to the enhanced rate capability; the increased low valence state V^4+^ may improve the conductivity of V-V_2_O_5_ and decrease electrochemical reaction resistance. Furthermore, the results indicated that the synergistic effect of V^4+^ and oxygen vacancy will improve the structure integrity and enhance the diffusion of Li ion. It enlightens us that adjusting the proportion of different valance state of metal elements in metallic oxides is a promising approach for improving their lithium storage for LIBs.

## Conflicts of interest

There are no conflicts to declare.

## Supplementary Material

## References

[cit1] Kim T., Shin J., You T. S., Lee H., Kim J. (2015). Thermally controlled V_2_O_5_ nanoparticles as cathode materials for lithium-ion batteries with enhanced rate capability. Electrochim. Acta.

[cit2] McNulty D., Buckley D. N., O'dwyer C. (2014). Synthesis and electrochemical properties of vanadium oxide materials and structures as Li-ion battery positive electrodes. J. Power Sources.

[cit3] Wang Y., Cao G. Z. (2006). Synthesis and enhanced intercalation properties of nanostructured vanadium oxides. Chem. Mater..

[cit4] Huang X., Rui X. H., Hng H. H., Yan Q. Y. (2015). Vanadium pentoxide-based cathode materials for lithium-ion batteries: Morphology control, carbon hybridization, and cation doping. Part. Part. Syst. Charact..

[cit5] Pan A. Q., Wu H. B., Zhang L., Lou X. W. (2013). Uniform V_2_O_5_ nanosheet-assembled hollow microflowers with excellent lithium storage properties. Energy Environ. Sci..

[cit6] Li Y. W., Yao J. H., Uchaker E., Zhang M., Tian J. J., Liu X. Y., Cao G. Z. (2013). Sn-doped V_2_O_5_ film with enhanced lithium-ion storage performance. J. Phys. Chem. C.

[cit7] Huang J. F., Qiao X. N., Xu Z. W., Ouyang H. B., Li J. Y. (2015). V_2_O_5_ nanoflowers assembled by nanorods as cathode material for lithium-ion batteries. Micro Nano Lett..

[cit8] Wu H. Y., Qin M. L., Li X. L., Cao Z. Q., Jia B. R., Zhang Z. L., Zhang D. Y., Qu X. H., Volinsky A. A. (2016). One step synthesis of vanadium pentoxide sheets as cathodes for lithium ion batteries. Electrochim. Acta.

[cit9] Yu H., Rui X., Tan H., Chen J., Huang X., Xu C., Liu W., Yu D. Y., Hng H. H., Hoster H. E. (2013). Cu doped V_2_O_5_ flowers as cathode material for high-performance lithium ion batteries. Nanoscale.

[cit10] Shin J., Jung H., Kim Y., Kim J. (2014). Carbon-coated V_2_O_5_ nanoparticles with enhanced electrochemical performance as a cathode material for lithium ion batteries. J. Alloys Compd..

[cit11] Tian S., Xing A., Tang H., Bao Z. H., Wu G. M. (2014). Enhanced cycling stability of TiO-coated V O nanorods through a surface sol-gel process for lithium ion battery applications. J. Mater. Chem. A.

[cit12] Song H. Q., Liu C. F., Zhang C. K., Cao G. Z. (2016). Self-doped V^4+^-V_2_O_5_ nanoflake for 2 Li-ion intercalation with enhanced rate and cycling performance. Nano Energy.

[cit13] Peng X., Zhang X. M., Wang L., Hu L. S., Cheng S. H. S., Huang C., Gao B., Ma F., Huo K. F., Chu P. K. (2016). Hydrogenated V_2_O_5_ nanosheets for superior lithium storage properties. Adv. Funct. Mater..

[cit14] Liu D. W., Liu Y. Y., Pan A. Q., Nagle K. P., Seidler G. T., Jeong Y. H., Cao G. Z. (2011). Enhanced Lithium-Ion Intercalation Properties of V2O5 Xerogel Electrodes with Surface Defects. J. Phys. Chem. C.

[cit15] Demeter M., Neumann M., Reichelt W. (2000). Mixed-valence vanadium oxides studied by XPS. Surf. Sci..

[cit16] Benayad A., Martinez H., Gies A. (2006). XPS investigations achieved on the first cycle of V_2_O_5_ thin films used in lithium microbatteries. J. Electron Spectrosc. Relat. Phenom..

[cit17] Liu D. W., Liu Y. Y., Garcia B. B., Zhang Q. F., Pan A. Q., Jeong Y. H., Cao G. Z. (2009). V_2_O_5_ xerogel electrodes with much enhanced lithium-ion intercalation properties with N_2_ annealing. J. Mater. Chem..

[cit18] Gao X. T., Zhu X. D., Le S. R., Yan D. J., Qu C. Y., Feng Y. J., Sun K. N., Liu Y. T. (2016). Boosting High-Rate Lithium Storage of V_2_O_5_ Nanowires by Self-Assembly on N-Doped Graphene Nanosheets. ChemElectroChem.

[cit19] Jia G. Q., Deng Z. N., Liu X., Jiang H., Li C. Z. (2016). Building radially oriented architecture by tailorable V_2_O_5_ nanoribbons toward enhanced lithium storage. Chem. Eng. J..

[cit20] PrześniakWelenc M., Karczewski J., SmalcKoziorowska J., Łapiński M., Sadowski W., Kościelska B. (2016). The influence of nanostructure size on V_2_O_5_ electrochemical properties as cathode materials for lithium ion batteries. RSC Adv..

[cit21] Li Y. W., Yao J. H., Uchaker E., Yang J. W., Huang Y. X., Zhang M., Cao G. Z. (2013). Leaf-like V_2_O_5_ nanosheets fabricated by a facile green approach as high energy cathode material for lithium-ion batteries. Adv. Energy Mater..

[cit22] Zhu C. C., Shu J., Wu X. Z., Li P., Li X. (2015). Electrospun V_2_O_5_ micro/nanorods as cathode materials for lithium ion battery. J. Electroanal. Chem..

